# Classical and Emerging Biomarkers in Pyridoxine-Dependent Epilepsy (PDE-ALDH7A1): Implications for Early Diagnosis and Therapeutic Development

**DOI:** 10.3390/biom16040486

**Published:** 2026-03-24

**Authors:** Muna Abedrabbo, Safiya Al Yazeedi, Blair R. Leavitt, Hilal Al-Shekaili

**Affiliations:** 1 Centre for Molecular Medicine and Therapeutics, BC Children’s Hospital and the Department of Medical Genetics, University of British Columbia, 950 West 28th Avenue, Vancouver, BC V5Z 4H4, Canada; mabedrabbo@cmmt.ubc.ca (M.A.); bleavitt@cmmt.ubc.ca (B.R.L.); 2 College of Medicine and Health Sciences, Sultan Qaboos University, P.O. Box 36, Muscat 123, Oman; safiya.alyazeedi@yahoo.com; 3 Incisive Genetics, 200–980 George Street, Vancouver, BC V6A 0H9, Canada; 4 Department of Biology, College of Science, Sultan Qaboos University, P.O. Box 36, Muscat 123, Oman

**Keywords:** pyridoxine-dependent epilepsy-ALDH7A1, α-aminoadipate semialdehyde (α-AASA), Δ^1^-piperideine-6-carboxylate (P6C), 6-oxopiperidine-2-carboxylic acid (6-oxo-PIP), 2-oxopropylpiperidine-2-carboxylic acid (2-OPP), 6-hydroxy-2-aminocaproic acid (HACA)

## Abstract

Pyridoxine-dependent epilepsy due to ALDH7A1 deficiency (PDE-ALDH7A1) is a rare but treatable epileptic encephalopathy caused by disruption of lysine catabolism and secondary depletion of pyridoxal-5′-phosphate (PLP). Although seizures are often controlled with pyridoxine supplementation, many patients continue to experience neurodevelopmental impairment, underscoring the importance of early diagnosis and improved therapeutic strategies. Central to both diagnosis and pathophysiology is the accumulation of lysine-derived metabolites, most notably α-aminoadipate semialdehyde (α-AASA), its cyclic Schiff base Δ^1^-piperideine-6-carboxylate (P6C), and pipecolic acid. These metabolites have become the biochemical hallmarks of PDE-ALDH7A1, linking *ALDH7A1* pathogenic variants to PLP inactivation and neuronal dysfunction. However, their chemical instability and analytical requirements pose challenges for universal diagnostics and newborn screening. This review summarizes current understanding of lysine catabolism in health and disease, critically evaluates the diagnostic utility and limitations of classical biomarkers, and discusses emerging insights into their pathophysiological roles. We further highlight recent discoveries of novel, chemically stable biomarkers, including 6-oxopiperidine-2-carboxylic acid (6-oxo-PIP), 2-oxopropylpiperidine-2-carboxylic acid (2-OPP), and 6-hydroxy-2-aminocaproic acid (HACA), identified through advanced metabolomics approaches. These metabolites show promise for newborn screening and provide new mechanistic links between metabolic stress, seizure susceptibility, and ongoing neurological morbidity despite pyridoxine treatment. Collectively, advances in biomarker discovery are reshaping diagnostic strategies for PDE-ALDH7A1 and offering new perspectives on disease mechanisms, paving the way for earlier detection and the development of more effective, mechanism-based therapies.

## 1. Introduction

Pyridoxine-dependent epilepsy (PDE-ALDH7A1, OMIM 266100) is an epileptic encephalopathy, characterized by pharmacoresistant seizures that are clinically and/or electroencephalographically responsive to pyridoxine (PN; a form of vitamin B6, vitB6) supplementation [1]. The first reported case of PDE-ALDH7A1 was in 1957, describing an infant with intractable convulsions unresponsive to standard antiepileptic treatment but successfully managed with a vitB6-containing multivitamin cocktail [2]. Since then, a broader clinical spectrum of PDE-ALDH7A1 has been recognized, ranging from the classical presentation of early-onset, treatment-resistant array of seizures (partial, myoclonic, clonic, and bilateral tonic–clonic seizures) to status epilepticus, often preceded by hyper-alertness, sleeplessness, feeding difficulties, and emesis [3,4,5,6].

Although pharmacological doses of PN are central to seizure control, with the required addition of antiseizure medications for optimal seizure control in some patient cases [1], approximately 70% of affected individuals exhibit developmental delay and intellectual disability, primarily affecting verbal abilities, despite adequate seizure control [3,7,8,9]. Neurologic manifestations include structural and neuropathological brain abnormalities, such as cerebral heterotopia, cortical malformations, hypoplasia of the corpus callosum, delayed myelination, and ventriculomegaly [7,10,11,12]. Additionally, lesions secondary to epilepsy, including hippocampal sclerosis, reactive gliosis, intracerebral hemorrhages, and white matter atrophy, have been observed [10,12]. Metabolic abnormalities associated with PDE-ALDH7A1 include depleted levels of PLP in cerebrospinal fluid (CSF) [13], hypoglycemia, lactic acidosis, electrolyte imbalances, coagulopathy, and altered plasma and CSF amino acid levels [3].

Although the current diagnostic pipeline for PDE mainly focuses on genetic testing and/or detection of specific biomarkers of the disease, prior to 2006, the underlying etiology of PDE-ALDH7A1 remained elusive for many years, and diagnosis was primarily based on the cessation of seizures after PN administration [1,14]. To confirm the diagnosis, this will be followed by treatment withdrawal, and if seizures relapse, then this indicates a true PN dependency [9]. Due to the distinct and robust response to high doses of PN, early hypotheses suggested a link between PDE-ALDH7A1 and vitB6 metabolism [15].

VitB6 is a general term that refers to a family of six interconvertible vitamers, namely pyridoxine (PN), pyridoxamine (PM), pyridoxal (PL), and their phosphorylated derivatives pyridoxine-5′-phosphate (PNP), pyridoxamine-5′-phosphate (PMP), and pyridoxal-5′-phosphate (PLP) [16]. Among these, PLP is the biologically active vitamer that acts as a cofactor for ~56–70 PLP-dependent proteins in the human body [3,16,17]. Early research posited that PDE-ALDH7A1 might result from defects in PLP-dependent enzymes; however, genetic linkage studies demonstrated that such enzymatic deficiencies were not the primary cause of PDE-ALDH7A1 [15,18,19]. This led researchers to explore alternative mechanisms, particularly those affecting PLP stability and availability [16,20]. Given that PLP’s aldehyde group at the 4’ position is highly reactive and prone to spontaneous interactions with various cellular molecules, attention shifted towards factors influencing its homeostasis [16]. This culminated in the discovery of the molecular basis of PDE-ALDH7A1 in 2006 when autosomal recessive pathogenic variants in the *ALDH7A1* gene, encoding α-aminoadipate semialdehyde dehydrogenase (AASDH; E.C. 1.2.1.31), also known as antiquitin, were identified as the genetic cause of the disorder [20]. Antiquitin catalyzes the third step in the lysine catabolism pathway, and its deficiency leads to the pathological accumulation of lysine intermediates, ultimately disrupting PLP availability and resulting in the characteristic features of PDE-ALDH7A1 [20].

Lysine catabolism is cellularly compartmentalized into two distinct, yet convergent pathways, the mitochondrial saccharopine pathway and the peroxisomal/cytosolic pipecolic acid pathway (Graphic Abstract) [21]. The saccharopine pathway begins with the ε-deamination of lysine to saccharopine by lysine-ketoglutarate reductase (LKR; EC 1.5.1.8), followed by oxidation to α-aminoadipate semialdehyde (α-AASA) and glutamate by saccharopine dehydrogenase (SDH; EC 1.5.1.9) [22,23]. α-AASA is found in equilibrium with its spontaneous cyclic form Δ1-piperideine-6-carboxilic acid (P6C) [20]. Both LKR and SDH are enzymatic domains of a single bifunctional mitochondrial enzyme known as aminoadipate semialdehyde synthase (AASS) [24]. AASDH (antiquitin) subsequently catalyzes the conversion of α-AASA into α-aminoadipic acid (α-AAA). α-AAA is then metabolized to 2-oxoadipic acid (OA) by the enzyme kynurenine/α-aminoadipic acid aminotransferase (AADAT). In the next step, 2-oxoadipic acid dehydrogenase complex (OADHc) carries out oxidative decarboxylation of OA to glutaryl-CoA. The latter undergoes a series of downstream reactions that culminate in the generation of acetyl-CoA which enters the tricarboxylic acid (TCA) cycle, completing the degradation process [21,25]. The last three steps of the lysine degradation pathway are identical to those of the final cycle of mitochondrial fatty acid β-oxidation pathway [13].

The alternative pipecolic acid pathway remains less well characterized [21]. Initial studies in rat and mouse brains suggested that lysine undergoes α-deamination to form pipecolic acid (PA), but the enzymes mediating the conversion of lysine to 2-oxo-6-aminocaproic acid (KAC) remain unidentified [26,27,28]. KAC spontaneously cyclizes to Δ1-piperideine-2-carboxylate (P2C), which is converted into PA by the cytosolic CRYM/ketamine reductase [29]. Further peroxisomal oxidation by pipecolic acid oxidase (PIPOX) generates P6C, the cyclic Schiff base of α-AASA, linking the two pathways at the antiquitin-catalyzed step [30].

*ALDH7A1* deficiency results in the pathological accumulation of lysine intermediate catabolites, including PA, α-AASA, and its Schiff base, P6C [20]. P6C readily condenses with and inactivates PLP, through a Knoevenagel condensation reaction, leading to PLP depletion [20]. Given the role of PLP as a cofactor in neurotransmitter metabolism, particularly in the synthesis of γ-aminobutyric acid (GABA), dopamine, and serotonin, its depletion has been implicated in PDE-ALDH7A1 seizure pathophysiology [15,31]. However, analysis of CSF neurotransmitter levels in PDE-ALDH7A1 patients have shown inconsistent results, indicating an incomplete understanding of the disease mechanism [32,33].

Currently, therapeutic paradigms aim to replenish PLP levels through PN supplementation and reduce neurotoxic lysine metabolites via a lysine-restricted diet [34]. While PN treatment effectively control seizures, the majority of patients continue to exhibit cognitive and developmental deficits [3,11]. Additionally, according to the online PDE-ALDH7A1 registry, breakthrough epileptic seizures might still occur despite the ongoing PN treatment (observed in 40% of the 116 registered patients) [34,35]. Moreover, a considerable proportion of patients remain in need of add-on anticonvulsants [34,35]. To overcome these limitations, in 2015, Coughlin et al. proposed a triple-therapy approach comprising PN supplementation, a lysine-restricted diet, and L-arginine supplementation [36]. Arginine competes with lysine for transport across the mitochondrial membrane and blood–brain barrier, thereby reducing lysine influx [36]. Although triple therapy significantly lowered plasma metabolite levels in some patients, its impact on CSF α-AASA levels was inconsistent, and cognitive outcomes showed only partial improvement [36]. This could be attributed partly to the neurodevelopmental impairments that occurred prior to diagnosis [11,14,37]. In addition, the pathophysiological mechanisms underlying these clinical observations remain incompletely recognized [35]. Recent evidence has focused on addressing the importance of diagnosis time. A more recent study by Coughlin et al. showed in a multicenter study that included 60 patients that a lysine-restricted diet, arginine supplementation, or a combination of both increased mean developmental test scores by 6.9 points overall, but when any of these diets were started within the first six months, the gain rose to ~22 points when compared with pyridoxine treatment alone [38]. Moreover, Footitt et al. were able to demonstrate that patients who started on lysine-restricted diet before they were six months old quickly reached target plasma lysine levels, with a significant drop in their urinary α-AASA levels. More importantly their cognitive scores remained stable or improved, compared to patients who started the diet later [39]. This evidence suggests that diagnostic delay poses a significant challenge that contribute to disease morbidity.

Therefore, a deeper understanding of PDE-ALDH7A1 biomarkers and their pathophysiological roles is essential for improving early diagnosis and devising more effective therapeutics against neurodevelopmental disabilities. This review will discuss recent advancements in PDE-ALDH7A1 biomarkers and their implications for early diagnosis through newborn screening and treatment strategies.

## 2. Classical Diagnostic Biomarkers

### 2.1. The Interplay Between the Saccharopine and Pipecolic Acid Pathways of Lysine Catabolism

Disentangling the relationship between the saccharopine and pipecolic acid pathways of lysine catabolism is critical in understanding the biochemical basis of PDE-ALDH7A1. Historically, the two pathways of lysine catabolism were thought to be spatially and temporally separated. The mitochondrial saccharopine pathway was regarded as the predominant route for lysine catabolism in the liver, kidney, and fetal brain [28]. In contrast, the adult mammalian brain was believed to rely mainly on the peroxisomal/cytosolic pipecolic acid pathway, with merely a minor saccharopine contribution [28]. This understanding was supported by early enzymatic studies along with ^14^C-lysine tracing experiments of radioactive lysine metabolites in rat brain and liver. These studies reported high levels of L-pipecolate in rat brain following injection of labeled lysine, while extracerebral tissues displayed an opposite pattern [26,40]. However, this notion has been more recently challenged by quantitative mass spectrometry analysis of lysine catabolites following IP injection of α- and ε-^15^N-labelled lysine into adult wildtype mice. This differential ^15^N labeling of the amine nitrogen in lysine provided a more accurate tracing of its metabolites since it can discriminate between the saccharopine and the pipecolic acid pathways. Lysine flux analysis of injected mice demonstrated that the saccharopine pathway is the one responsible for supplying the bulk of α-AAA in the liver, kidney, and even in cerebral tissue. On the other hand, pipecolic acid pathway did not significantly contribute to the local and systemic levels of α-AAA [41]. This was further supported by another flux analysis in mouse primary astrocytes and HEK293 cells using ^15^N as a tracer. In this study, labeling was only detected from α-^15^N-lysine- and not ε-^15^N-lysine-derived α-AAA, confirming that the saccharopine pathway is primarily active in both cell types [42]. Collectively, these newer data revise the classic organ-specific paradigm, positioning the saccharopine pathway as the central lysine-catabolic route across most tissues, including the adult brain, while the pipecolic acid pathway appears to play a comparatively minor, perhaps regulatory, role.

The next two sub-sections will delve deeper into the biochemical and pathophysiological features of α-AASA, P6C, and PA in PDE-ALDH7A1, exploring how these metabolites contribute to disease pathology and the challenges associated with their utility in clinical diagnostics.

### 2.2. Diagnostic Advances and Limitations of Traditional Biomarkers

#### 2.2.1. Pipecolic Acid

Early studies identified elevated levels of PA in the plasma and CSF of patients with PDE-ALDH7A1, linking it to the disruption of lysine metabolism. In 2000, a study conducted by Plecko, B. et al. reported on the isolated elevation of PA in plasma and/or CSF in two patients with PDE-ALDH7A1 with an inverse correlation of PA concentrations in plasma and/or CSF after the oral intake of PN [43]. The same group later confirmed elevated PA in plasma, CSF and/or urine as a diagnostic marker in six additional PDE-ALDH7A1 patients [44]. However, in a subsequent study, they highlighted the limitations of PA as a diagnostic marker, demonstrating that its elevation in PDE-ALDH7A1 was only moderate compared to controls and it was expressed in a wide range of concentrations in sampled patients. Moreover, these PA levels also overlap with levels seen in other peroxisomal metabolic disorders such as Zellweger syndrome, due to impaired peroxisomal fatty acid oxidation [45,46]. Other reports describe PDE-ALDH7A1 patients whose plasma PA fall within the normal reference range, demonstrating that normal plasma PA levels do not exclude PDE-ALDH7A1 diagnosis [47,48]. Thus, its lack of disease-specificity and non-consistent elevation among patients diminish its diagnostic utility in PDE-ALDH7A1.

In an effort to understand its precise biochemical relationship with PDE-ALDH7A1, Mills et al. postulated, in their seminal *ALDH7A1* discovery paper, that PA accumulation in CSF and plasma of PDE-ALDH7A1 may result directly from the pipecolic acid pathway, which was thought to be the main pathway of lysine catabolism in cerebral tissue [20,28]. However, more recent mechanistic work using isotopic tracking in *ALDH7A1*-deficient fibroblasts showed that PA was derived from the saccharopine pathway and postulated that P6C is the direct precursor for the retrograde formation of PA [49]. In a subsequent study, it was shown that the mitochondrial pyrroline-5-carboxylate reductase (PYCR1) is the enzyme responsible for the conversion of saccharopine pathway-derived P6C to PA, providing an alternative source of PA that is independent of the canonical pipecolic acid pathway [49,50]. Thus, the rise in PA levels in PDE-ALDH7A1 patients may reflect mitochondrial PYCR1 activity on accumulated P6C rather than a simple compensatory flux through the pipecolic acid pathway [49,50]. This dual origin explains why PA, while a useful marker, is not entirely specific for the pipecolic acid pathway. This, along with its non-specificity to PDE-ALDH7A1, underscore the need to combine it with more definitive biomarkers for PDE-ALDH7A1 diagnosis.

An important consideration to take into account when interpreting PA measurements as a biomarker for PDE-ALDH7A1 is the circulating levels coming from intestinal microbiota. Many lines of evidence demonstrate that PA can be produced from dietary lysine by gut bacteria. Studies by Schmidt-Glenewinkel et al. demonstrated that conversion of lysine to piperidine (a metabolite of pipecolic acid) occurred exclusively in the intestines and was attributed to intestinal flora activity [51]. Furthermore, studies using lysine loading tests revealed that the D-isomer of PA, which increased significantly following dietary lysine intake, originates predominantly from catabolism of dietary lysine by intestinal bacteria rather than from direct food intake, while the L- and D-isomers appear to have different metabolic mechanisms [52]. These findings indicate that when using pipecolic acid as a diagnostic or monitoring biomarker in pyridoxine-dependent epilepsy, clinicians should consider potential confounding effects of gut microbiota composition, antibiotic use, and dietary lysine intake on measured pipecolic acid concentrations.

#### 2.2.2. α-AASA and P6C

The discovery of α-AASA as a diagnostic biomarker for PDE-ALDH7A1 has been pivotal in understanding the biochemical and genetic basis of this disorder. In 2006, Mills et al. were the first to identify α-AASA as a key metabolite accumulating in patients with *ALDH7A1* pathogenic variants [20]. Using liquid chromatography–tandem mass spectrometry (LC-MS/MS), they demonstrated significantly elevated levels of α-AASA in urine, plasma, and cerebrospinal fluid (CSF) of PDE-ALDH7A1 patients compared to controls [20]. This disruption in lysine catabolism provided the first biochemical explanation for the seizures and neurological deficits observed in PDE-ALDH7A1. Mills et al. also established that urinary α-AASA measurement offers a non-invasive and reliable diagnostic method, making it a preferred biomarker over PA, which may require invasive CSF sampling and is less specific [20]. Similarly, Bok et al. reported elevated urinary α-AASA levels in Dutch patients with PDE-ALDH7A1, using LC-MS/MS for quantification [53]. Their findings emphasized the utility of this metabolite in routine diagnostic workup of PDE-ALDH7A1 [53]. A year later, Plecko et al. conducted a comprehensive biochemical and molecular analysis of 18 PDE-ALDH7A1 patients, further validating the role of α-AASA as a diagnostic marker [45]. Using LC-MS/MS, they confirmed the consistent elevation of α-AASA in urine and identified various *ALDH7A1* pathogenic variants, linking these genetic defects to the biochemical phenotype [45]. The study also highlighted the importance of α-AASA in differentiating PDE-ALDH7A1 from other metabolic disorders, such as peroxisomal diseases, in which PA is elevated but α-AASA remains within normal levels. Subsequent case reports described elevated urinary α-AASA in patients affected with molybdenum cofactor or sulfite oxidase deficiencies [54]. Of note here, these cases may also present with PN-responsive seizures.

P6C, the cyclic form of α-AASA, plays a central role in the pathophysiology of PDE-ALDH7A1. Mills et al. demonstrated that P6C accumulates due to antiquitin deficiency and subsequently inactivates PLP through a Knoevenagel condensation reaction [20]. Using LC-MS/MS, they detected the formation of condensation products between P6C and PLP, providing mechanistic insights into the disease [20]. This inactivation of PLP, a crucial cofactor for neurotransmitter synthesis, is believed to be the main driver of the epileptic seizures observed in PDE-ALDH7A1 [20]. The study also highlighted the equilibrium between α-AASA and P6C, explaining their co-accumulation in body fluids [20]. Following this landmark study, Sadilkova et al. reported elevated levels of P6C in plasma samples from a new cohort of PDE-ALDH7A1 patients, further supporting its diagnostic potential [48]. In an effort to establish less invasive methods for detection, Struys et al. were capable of identifying elevated levels of P6C in urine samples of 35 PDE-ALDH7A1 patients using a semi-quantitative LC-MS/MS method, concluding that the diagnostic power of urinary P6C and α-AASA are comparable [55]. Together, these studies have established α-AASA and P6C as the gold standards for PDE-ALDH7A1 diagnosis, providing a clear biochemical link between *ALDH7A1* pathogenic variants, lysine pathway disruption, and disease pathology.

### 2.3. The Roles of α-AASA, P6C, and Pipecolic Acid in the Pathophysiology of PDE-ALDH7A1

Following the identification of the genetic basis of PDE-ALDH7A1, subsequent research has focused on defining the pathophysiological roles of the accumulating lysine-derived metabolites, namely P6C, α-AASA, and pipecolic acid.

Multiple factors contribute to the toxicity of α-AASA and P6C. The earliest evidence for P6C toxicity came from the nature of its interaction with PLP, a cofactor required for ~70 enzymatic reactions including those involved in the metabolic pathways of important neurotransmitters like GABA [17,56,57,58]. Since GABA is the principal inhibitory neurotransmitter in the CNS, its deficiency may disrupt the excitatory/inhibitory balance in the brain and subsequently predispose to epileptic seizures [59]. The GABA hypothesis was first put forward in 1960 by Scriver who suggested that dysfunction of glutamic acid decarboxylase, a PLP-dependent key enzyme in the production of GABA, is a potential contributing cause of seizures in PDE-ALDH7A1 [60]. This hypothesis was supported by a 1994 report of reduced GABA levels in fibroblasts derived from PDE-ALDH7A1 patients [15]. However, measurements of GABA and other neurotransmitters in CSF samples from PDE patients have shown inconsistent results, leaving their role in the pathophysiology of PDE-ALDH7A1 incompletely defined [61].

More recently, exposure of mouse neural progenitor cells (NPCs) to supraphysiological concentrations of α-AASA/P6C inhibited NPC proliferation and neuronal differentiation in vitro [62]. The same study showed that α-AASA/P6C reduced the expression of genes governing de novo pyrimidine biosynthesis, leading to abnormal hippocampal neurogenesis and cognitive impairment in *Aldh7a1*-deficient adult mice, thereby linking α-AASA/P6C to the intellectual disability observed in patients [62]. Subsequent work by the same group examined astrocyte-derived α-AASA/P6C and its impact on dendritic spine development [63]. The authors concluded that dendritic spine impairment was not mediated by α-AASA/P6C accumulation per se, but by an astrocyte-secreted extracellular matrix molecule [63]. However, it is still plausible that accumulation of α-AASA/P6C in astrocytes may disrupt neurotransmitter regulation and potassium uptake from extracellular space, potentially contributing to aberrant synaptic transmission [63,64].

Beyond these roles, it has been hypothesized that, due to their reactive chemical nature, α-AASA and/or P6C can induce oxidative stress in neurons and astrocytes via different mechanisms like protein carbonylation and lipid peroxidation [65]. Oxidative damage further compromises mitochondrial function and may trigger neurotoxic cascades [65].

Similarly, since PA catabolism can produce hydrogen peroxide by oxidases, emerging evidence has underscored the role of PA in oxidative stress, mitochondrial dysfunction, and the activation of apoptotic pathways in neuronal cells. In a 2014 study, Dalazen et al. demonstrated that incubation of rat cerebral cortex supernatants with PA for one hour resulted in decreased activity of antioxidant enzymes that was rescued by the addition of an efficient antioxidant [66]. Further investigations with mouse neuroblastoma cells revealed that treatment with both D- and L-pipecolate for over 72 h resulted in reduced cell viability, with a racemic mixture of D- and L-pipecolate demonstrating the highest toxicity [67].

Beyond these toxic effects, PA has also been demonstrated to possess neuroactive properties. The seminal studies by Giacobini’s group in the 1970s and 1980s established several critical aspects of pipecolic acid biochemistry in the CNS [68]. Schmidt-Glenewinkel et al. [51] demonstrated that lysine is converted to pipecolic acid in brain tissue, and subsequent work by Nishio and Giacobini characterized the BBB transport of pipecolic acid [69]. Their research further demonstrated that pipecolic acid accumulates rapidly in brain tissue, exhibits saturable uptake by synaptosomes [70], and can be released from hippocampal slices in a calcium-dependent manner following potassium-induced depolarization [71], suggesting a potential neuromodulatory role.

Later, PA interactions with the GABAergic system were described. Gutiérrez and Delgado-Coellodemonstrated that physiological concentrations of PA increased potassium-stimulated release of [^3^H]GABA in mouse cerebral cortex slices and significantly diminished GABA uptake, suggesting that PA acts primarily at the presynaptic level to modulate GABAergic neurotransmission [72]. This neuromodulatory activity may contribute to the pathophysiology of seizures in PDE-ALDH7A1, as elevated PA levels could dysregulate inhibitory neurotransmission.

In summary, the cumulative impact of these metabolites likely underlies the persistent developmental delay and cognitive impairment observed in PDE-ALDH7A1 patients. Nevertheless, further investigations are required to delineate the pathophysiological pathways implicated in the disease.

### 2.4. Model System-Derived Metabolites Relevant to PDE-ALDH7A1 Pathophysiology

Although saccharopine, an adduct of lysine and alpha-ketoglutarate, is not the immediate upstream metabolite of the antiquitin-catalyzed reaction, and has not been reported as a known biomarker in patients with PDE-ALDH7A1, elevated saccharopine levels have been reported in cellular, mouse, and zebrafish models of PDE-ALDH7A1 [42,61,73,74]. Johal et al. demonstrated marked saccharopine accumulation in the liver and kidney of adult *Aldh7a^−/−^* mice, as well as in primary astrocytes derived from these animals [42]. Additionally, Al-Shekaili et al. reported saccharopine accumulation in the brains of P0 *Aldh7a1^−/−^* mice [73]. Van Karnebeek et al. further showed saccharopine accumulation in both the brain and liver tissues of *Aldh7a1^−/−^* single knockout mice, a phenotype that was rescued in the *Aldh7a1/Aass* double-knockout model [74].

The pathological consequences of saccharopine accumulation have been investigated in models of hyperlysinemia type II (saccharopinuria; OMIM: 268700), a disorder characterized by defects in the SDH domain of the bifunctional enzyme AASS [75]. Zhou et al. reported that significant saccharopine accumulation in SDH-mutant *C. elegans* model disrupted mitochondrial dynamics manifesting as abnormal mitochondrial enlargement [76]. This disruption was proposed to arise from continuous mitochondrial fusion and impaired endoplasmic reticulum-dependent tubulation and fission, ultimately leading to mitochondrial damage and loss of function [76]. Consistent with these findings, introduction of an SDH mutation (Aass-G489E) in a mouse model led to hepatic mitochondrial damage and functional impairment, accompanied by developmental delay and premature death in homozygous mutant mice [76]. In a subsequent study, the same group investigated the impact of saccharopine accumulation in the brain using the same knock-in mouse model (Aass-G489E) and compared it to LKR-mutant (Aass-R65Q) mice that do not accumulate saccharopine [77]. Aass-G489E homozygous mutant mice exhibited reduced brain size and impaired dendritic arborization of pyramidal neurons in the cerebral cortex, a phenotype that was not observed in mice harboring the Aass-R65Q mutation [77]. Further mechanistic investigation using mass spectrometry revealed that saccharopine binds to and inhibits the cytokine activity of extracellular glucose-6-phosphate isomerase (GPI) [77]. GPI is a multifunctional protein that functions intracellularly in a dimeric form as a glycolytic enzyme and extracellularly in a monomeric form as a neuroleukin promoting neuronal survival [78,79]. Altogether, these studies provide novel mechanistic insight into the potential role of saccharopine accumulation as a contributor to the pathophysiology of PDE-ALDH7A1, although further work is needed to elucidate its actual levels in patients and whether this concurs with its reported buildup in cellular and animal models.

Despite the significant advancements in the validation and detection methods, traditional biomarkers like α-AASA and P6C continue to have limitations. The measurement of α-AASA and P6C requires advanced analytical techniques such as LC-MS/MS, which may not be universally available. Additionally, α-AASA levels are found to be unstable and can be influenced by sample handling and storage conditions, necessitating strict protocols to ensure accuracy [35,55]. This highlights the need for a broader diagnostic approach for PDE-ALDH7A1. The development of stable, non-invasive, and universally accessible diagnostic biomarkers remains a critical goal in improving the early detection and management of PDE-ALDH7A1. The next section will highlight two newly discovered biomarkers that hold promise in overcoming the limitations associated with traditional biomarkers.

## 3. Novel Discovered Biomarkers

### 3.1. 6-oxo-PIP and 2-OPP

Despite the promising therapeutic potential for PDE-ALDH7A1 [42,64,74], one of the major challenges contributing to disease morbidity is the potential risk of irreversible neurological impairments associated with delayed diagnosis [11,14]. Consequently, substantial efforts have been directed towards developing a newborn screening workflow for PDE-ALDH7A1 to ensure better neurodevelopmental outcomes [11,14,35]. This is particularly crucial given the relatively high incidence rate of PDE-ALDH7A1 (approximately 1:64,000 conceptions) [80]. However, previous attempts at newborn screening have been hindered by the limited thermal stability of the classical PDE-ALDH7A1 biomarkers (α-AASA, P6C, and PA) in urine, plasma, and dried blood spots (DBS), with the latter method being routinely used for newborn screening [14,35]. Furthermore, standard newborn screening workflows employ direct-infusion mass spectrometry without liquid chromatography-based metabolite separation to enable high-throughput analysis, necessitating the use of stable metabolites without derivatization steps [14,35]. Given these constraints, there has been an evident clinical need to identify new biomarkers that overcome the preanalytical stability challenges posed by the classical PDE-ALDH7A1 biomarkers.

In 2019, Wempe et al. attempted to enhance the stability of α-AASA for newborn screening by synthesizing α-AASA/P6C in vitro, followed by derivatization with 2,4-dinitrophenylhydrazine (2,4-DNP) [14]. This approach aimed to stabilize the aldehyde functional group of α-AASA and create a stable adduct detectable via MS; however, no reaction product was observed [14]. Subsequent attempts to characterize the in vitro product using nuclear magnetic resonance (NMR) spectroscopy, which provides detailed chemical structure information, led to uncovering of a P6C/6-hydroxypipecolate (6-OH-PIP) mixture in a rapid equilibrium [14]. The authors proposed that this mixture is a result of an intermediate step between α-AASA and P6C, in which α-AASA cyclizes without the loss of water to produce 6-OH-PIP, which subsequently dehydrates to form P6C (Graphic Abstract) [14].

To investigate this pathway further, they incubated the P6C/6-OH-PIP mixture with human liver cytosol, leading to the formation of α-AAA and small amounts of a newly identified metabolite, 6-oxopiperidine-2-carboxylic acid (6-oxo-PIP) [14]. To validate the presence of 6-oxo-PIP in PDE-ALDH7A1 patients, DBS and urine samples from patients and controls were analyzed using a targeted LC-MS/MS approach [14]. Unlike P6C/6-OH-PIP, which was rapidly degraded to undetectable levels, 6-oxo-PIP exhibited significantly higher concentrations in PDE-ALDH7A1 patients compared to controls, remaining quantifiable in initial DBS samples and persisting for up to four months in urine [14]. These findings underscore the superior stability of 6-oxo-PIP as a novel biomarker for PDE-ALDH7A1 [14].

Regarding the metabolic origins of 6-oxo-PIP, two potential sources have been proposed. First, given that 6-oxo-PIP is the cyclic lactam of α-AAA, direct cyclization from α-AAA is chemically feasible [14,81,82]. Wempe et al. noted that enzymatic formation of 6-oxo-PIP from α-AAA has been reported in *Penicillium chrysogenum* and observed small amounts of 6-oxo-PIP formation in their cytosol incubations [14]. Böhm et al. further proposed that this cyclization could occur via a 6-exo-trig mechanism according to Baldwin’s rules of cyclization, and they theoretically suggested that the reverse reaction (ring opening of 6-oxo-PIP to form α-AAA) might also be possible [81]. However, this hypothesis regarding reversibility is not supported by experimental evidence. Wempe et al. specifically tested this by incubating 6-oxo-PIP with human liver cytosol, S9 fractions, and human plasma, and found that none of these experiments resulted in the conversion of 6-oxo-PIP to α-AAA [14]. This demonstrates that while α-AAA can cyclize to form 6-oxo-PIP, the reverse hydrolysis of the lactam does not occur under physiological conditions. The second proposed source is the oxidation of 6-OH-PIP to 6-oxo-PIP by a cytosolic dehydrogenase, though the specific enzyme remains unidentified [14] (Graphic Abstract).

Since then, advancements in analytical techniques have offered new opportunities for identifying novel biomarkers for metabolic disorders, including PDE-ALDH7A1 [83]. While targeted LC-MS/MS enables sensitive and quantitative analysis of predefined metabolites, next-generation untargeted-metabolomics provides a semiquantitative, global detection approach that allows for the discovery of previously unrecognized biomarkers [83]. This approach was integrated by Engelke et al. in combination with infrared ion spectroscopy (IRIS), a structural elucidation technique, to identify novel biomarkers for PDE-ALDH7A1 [35]. Their study confirmed the previous novel biomarker, 6-oxo-PIP, and identified a new metabolite, 2S,6S-/2S,6R-oxopropylpiperidine-2-carboxylic acid (2-OPP), both served as discriminative biomarkers for PDE-ALDH7A1 [35]. The accumulation of these metabolites was validated in plasma and urine of untreated PDE patients using quantitative targeted assays for both metabolites, and they also accumulated in patients receiving PN supplementation [35]. These findings suggest that these metabolites may contribute to the neurodevelopmental phenotype in PDE-ALDH7A1 despite PN treatment [35]. Further evidence supporting the pathophysiological relevance of these metabolites was provided by their detection in a postmortem brain sample from a PDE-ALDH7A1 patient as well as in *Aldh7a1*-knockout mouse brain [35,73]. Notably, these metabolites exhibited a remarkable stability since they remained measurable in DBS samples stored at room temperature for 12–13 years [35].

The mechanism of 2-OPP production was subsequently investigated. Engelke et al. hypothesized that 2-OPP could be formed spontaneously via the interaction of P6C with acetoacetate under physiological conditions [35]. Incubating P6C with acetoacetate in different matrices resulted in the non-enzymatic formation of 2-OPP [35]. Given that acetoacetate levels usually increase during ketosis, which may not apply to all PDE-ALDH7A1 patients, they explored its relevance to non-ketotic PDE-ALDH7A1 patients and found that even low levels of acetoacetate were sufficient to facilitate the formation of 2-OPP [35].

Beyond biomarker stability for newborn screening, another challenge in the PDE-ALDH7A1 field is the incomplete understanding of the role of accumulating metabolites in the pathophysiology of the disease, specifically their potential contribution to seizures [11,35]. This is particularly relevant given reports of breakthrough seizures in PDE-ALDH7A1 patients despite PN supplementation [34,35]. Consequently, to investigate the neurotoxic potential of 2-OPP, Engelke et al. examined its effects on zebrafish larvae [35]. They observed that 2-OPP-treated larvae displayed seizure-like hyperactive behavior characterized by whole-body convulsions, leading to loss of posture in light/dark behavioral assay [35]. This phenotype is consistent with the seizure-like hyperactive phenotype described in *aldh7a1*-knockout zebrafish reported by Pena et al., suggesting that 2-OPP exerts neurotoxic effects [61]. These findings provide a possible mechanistic explanation for the occurrence of breakthrough seizures in PDE-ALDH7A1 patients during febrile illness despite PN treatment. Such episodes of illness may induce catabolic stress leading to increased flux through ketosis as well as amino acid metabolism, which might in turn produce higher levels of 2-OPP [35].

More recent evidence to support the utility of these two novel biomarkers in newborn screening came from a feasibility study that demonstrated the detectability of both metabolites in newborn DBS with the equipment already used in population-based screening. The authors reported detecting elevated levels of 2-OPP in 7/8 PDE-ALDH7A1 DBS samples using a first-tier flow-injection analysis-MS/MS assay, as well as 6-oxo-PIP in all samples using a second-tier LC-MS/MS run compared to controls [84]. A previous urinary biomarker study confirmed that 6-oxo-PIP is reliably elevated in patients older than six months, but it may be normal in infants < 6 months, limiting its usefulness as a primary newborn screen [85]. However, it is important to emphasize that this assessment was based on urine samples rather than dried blood spots (DBS), which are the standard matrix used in newborn screening workflows. Thus, the combinatorial detection of 6-oxo-PIP and 2-OPP from DBS remain a promising approach for newborn screening. Nevertheless, more studies are needed to determine the specificity and sensitivity of both biomarkers in neonatal DBS.

### 3.2. HACA

In 2022, global metabolomics performed by Böhm et al. also uncovered two PN-independent biochemical markers: 6-hydroxy-2-aminocaproic acid (HACA) and a less established C_9_H_11_NO_4_ isomer [81]. HACA showed a six-fold increase in patient plasma compared to non-PDE-ALDH7A1 controls and remained robust after 1.5 years when stored at −20 °C, with a clear linear correlation to PA [81]. The authors suggested that HACA might result from the non-enzymatic equilibrium with PA through a spontaneous cyclization with the loss of water. The exhibited increased stability and significant elevation of 2-OPP, 6-oxo-PIP, and HACA make them promising candidates for newborn screening assays [81].

## 4. Conclusions

In conclusion, classical biomarkers, α-AASA, P6C, and PA, have long anchored diagnosis and remain clinically indispensable, but their chemical instability and analytical requirements have always been a practical barrier, particularly for newborn screening where speed and reliability are non-negotiable. The identification of novel PDE-ALDH7A1 biomarkers represents a significant advancement not only for early diagnosis but also for deepening our understanding of disease pathophysiology. The discovery of 6-oxo-PIP, 2-OPP, and HACA has provided important insights into the molecular mechanisms of PDE-ALDH7A1. The fact that they persist in patients on pyridoxine raises the possibility that they may be active contributors to the ongoing neurological injury that pyridoxine alone cannot fully prevent. The seizure-like behavior observed in 2-OPP-exposed zebrafish larvae lends biological weight to that concern and reinforces the case for therapeutic strategies that go beyond vitamin supplementation to directly reduce toxic metabolite accumulation. What remains to be explored is substantial: larger prospective validation studies in neonatal dried blood spots, clearer mechanistic resolution of how these novel metabolites form and how they participate to disease pathology, and the development of biomarker panels specific enough to confidently distinguish PDE-ALDH7A1 from overlapping conditions. Converging research trajectories towards biomarker discovery, animal modeling, and emerging therapies will pave the way for better diagnostic strategies and more effective therapeutic interventions for patients.

## Data Availability

Not applicable.
